# The role of the *Acanthamoeba castellanii* Sir2-like protein in the growth and encystation of *Acanthamoeba*

**DOI:** 10.1186/s13071-020-04237-5

**Published:** 2020-07-22

**Authors:** So-Young Joo, Ja Moon Aung, Minsang Shin, Eun-Kyung Moon, Hyun-Hee Kong, Youn-Kyoung Goo, Dong-Il Chung, Yeonchul Hong

**Affiliations:** 1 Department of Parasitology and Tropical Medicine, School of Medicine, Kyungpook National University, Daegu, Republic of Korea; 2 Department of Microbiology, School of Medicine, Kyungpook National University, Daegu, Republic of Korea; 3 Department of Medical Zoology, Kyung Hee University School of Medicine, Seoul, Republic of Korea; 4 Department of Parasitology, Dong-A University College of Medicine, Busan, Republic of Korea

**Keywords:** *Acanthamoeba castellanii*, Encystation, Sirtuin-like protein, Salermide

## Abstract

**Background:**

The encystation of *Acanthamoeba* leads to the development of resilient cysts from vegetative trophozoites. This process is essential for the survival of parasites under unfavorable conditions. Previous studies have reported that, during the encystation of *A. castellanii*, the expression levels of encystation-related factors are upregulated. However, the regulatory mechanisms for their expression during the encystation process remains unknown. Proteins in the sirtuin family, which consists of nicotinamide adenine dinucleotide-dependent deacetylases, are known to play an important role in various cellular functions. In the present study, we identified the *Acanthamoeba* silent-information regulator 2-like protein (AcSir2) and examined its role in the growth and encystation of *Acanthamoeba.*

**Methods:**

We obtained the full-length sequence for AcSir2 using reverse-transcription polymerase chain reaction. In *Acanthamoeba* transfectants that constitutively overexpress AcSir2 protein, SIRT deacetylase activity was measured, and the intracellular localization of AcSir2 and the effects on the growth and encystation of trophozoites were examined. In addition, the sirtuin inhibitor salermide was used to determine whether these effects were caused by AcSir2 overexpression

**Results:**

AcSir2 was classified as a class-IV sirtuin. AcSir2 exhibited functional SIRT deacetylase activity, localized mainly in the nucleus, and its transcription was upregulated during encystation. In trophozoites, AcSir2 overexpression led to greater cell growth, and this growth was inhibited by treatment with salermide, a sirtuin inhibitor. When AcSir2 was overexpressed in the cysts, the encystation rate was significantly higher; this was also reversed with salermide treatment. In AcSir2-overexpressing encysting cells, the transcription of cellulose synthase was highly upregulated compared with that of control cells, and this upregulation was abolished with salermide treatment. Transmission electron microscope-based ultrastructural analysis of salermide-treated encysting cells showed that the structure of the exocyst wall and intercyst space was impaired and that the endocyst wall had not formed.

**Conclusions:**

These results indicate that AcSir2 is a SIRT deacetylase that plays an essential role as a regulator of a variety of cellular processes and that the regulation of AcSir2 expression is important for the growth and encystation of *A. castellanii*.
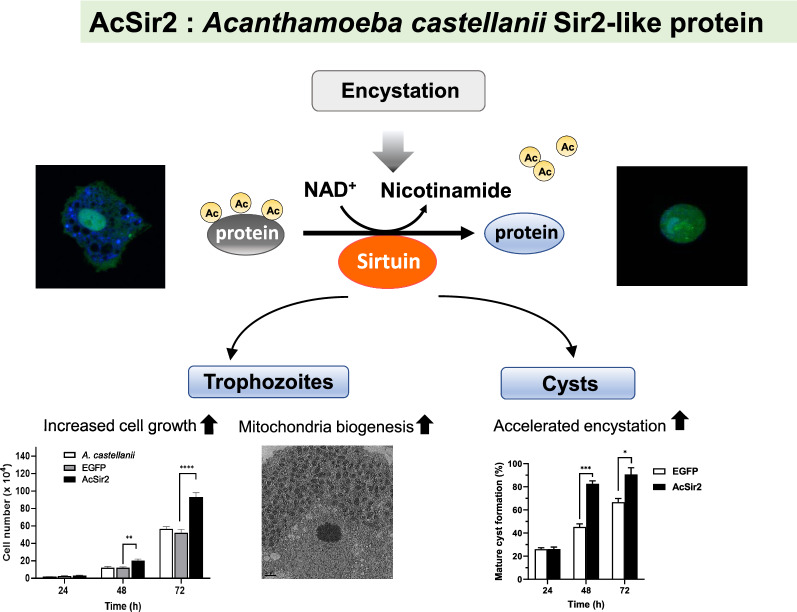

## Background

*Acanthamoeba*, an opportunistic human pathogen that causes granulomatous amoebic encephalitis, dermatitis, and amoebic keratitis, is widely distributed in both natural and artificial environments (see [1, 2] for a review). The life-cycle of *Acanthamoeba* has both trophozoites and cysts stages. Under conditions that are unfavorable for proliferation, such as a lack of nutrients, low temperatures, low moisture levels, conditions leading to hyperosmolarity, and the presence of biocides, vegetative *Acanthamoeba* trophozoites become metabolically inactive, dormant cysts *via* encystation [3–6]. *Acanthamoeba* cysts are double-walled and highly resistant to adverse conditions, thus prolonging their survival and facilitating their transmission [1, 3–8]. Thus, the inhibition of encystation during the treatment of amoebic infections can lead to more favorable outcomes. During the encystation process of *Acanthamoeba*, many encystation-related proteins, including cyst-specific protein (CSP21) [9], various proteases [10–13], autophagy-related factors [14–16], and cellulose synthase [17–20], have been shown to be upregulated and to play an important role in cyst formation. However, how the expression of encystation-related factors is controlled during the encystation process remains unclear.

Previously, Kong et al. [21] investigated encystation-mediating factors using the comparative microarray analysis of trophozoites and cysts. Using data mining of the DNA sequences of the upregulated genes in the encysting trophozoites, a cDNA fragment containing a member of the sirtuin family of proteins, which are transcription regulators, was found. Sirtuins are a silent-information regulator 2 (SIR2)-like family of protein deacetylases that require nicotinamide adenine dinucleotide (NAD) as a cofactor in the deacetylation reaction. Thus, it is generally believed that sirtuins act as a sensor of energetic status that interprets external changes and regulates internal responses at a subcellular level [22–24]. Sirtuins have been evolutionarily conserved from bacteria to humans. In humans, seven sirtuin family members (SIRT1 to SIRT7) have been reported, with each differing in terms of their subcellular localization and protein substrates [25]. SIRT1, SIRT6, and SIRT7 are found primarily in the nucleus, SIRT2 is localized in the cytosol, and SIRT3, SIRT4, and SIRTt5 are mitochondrial proteins. Given that they are found in different locations within a cell, sirtuins are involved a variety of critical cellular processes, including transcription, DNA repair, metabolism, and stress resistance [26, 27]. Additionally, sirtuins regulate the number, activity, and turnover of mitochondria [28].

Sirtuins have been positively associated with a longer life span in some organisms and the potential regulation of pathways mediated by nutrient starvation (see [29, 30] for a review). They have also been connected to numerous human diseases [31], thus sirtuin inhibitors have attracted significant attention as potential therapeutics [32]. In some parasites, sirtuins play a crucial role in the modulation of parasite gene expression, and a significant number are known to be crucial to the survival and/or development of several parasites under various conditions; as a result, this family of proteins has been identified as a potential target for the development of anti-parasitic therapies [33, 34]. For example, the sirtuin inhibitors sirtinol and salermide have been shown to interfere with the growth of *Leishmania infantum* [35] and *Trypanosoma cruzi* [36], respectively, while, in *Schistosoma mansoni*, some sirtuin inhibitors induce the apoptosis and death of schistosomula, the disruption of adult worm pairs, and a reduction in egg-laying [37, 38].

In the present study, we identify a sirtuin in *A. castellanii*, examine its role in trophozoite growth and encystation, and assess its potential as a therapeutic target in the treatment of *Acanthamoeba* infections.

## Methods

### *Acanthamoeba castellanii* cultures and the induction of encystation

The Neff strain of *Acanthamoeba castellanii* (ATCC #30010) was obtained from the American Type Culture Collection (Manassas, VA, USA) and cultured in peptone-yeast-glucose (PYG) medium at 25 °C, as previously described [39]. The encystation of *Acanthamoeba* was then induced as previously described with slight modifications [40]. Briefly, 1 × 10^6^*A. castellanii* cells in their post-logarithmic growth phase were collected and washed with phosphate-buffered saline (PBS) and incubated in 10 ml of encystment medium (95 mM NaCl, 5 mM KCl, 8 mM MgSO_4_, 0.4 mM CaCl_2_, 1 mM NaHCO_3_ and 20 mM Tris-HCl, pH 9.0) at 25 °C for 72 h. The morphological transformation of the cells into cysts was observed using light microscopy. The number of cells was counted after treating the cells with 0.4% trypan blue, which selectively stains non-viable cells [41]. Encystation efficiency was assessed by counting the number of cysts under a light microscope after treating the cells with 0.5% sodium dodecyl sulfate [42].

### Cloning, quantitative transcriptional profiling and phylogenetic analysis of AcSir2

The full-length cDNA sequence for *AcSir2* was isolated from AmoebaDB (AmoebaDB: ACA1_084880) [43] and verified using reverse transcription polymerase chain reaction (RT-PCR) using sequence-specific primers (sense: 5′-ATG GCC AGC ACA GTC GAC TC-3′; antisense: 5′-TTA GGC GAC GTC GAT CAG TT-3′). The expression of AcSir2 in both trophozoites and encysting cells were determined using quantitative real-time PCR (qRT-PCR). The total RNA of the *Acanthamoeba* trophozoites and cysts was purified using RNeasy Mini Kits following the manufacturer’s instructions (Qiagen, Hilden, Germany). qRT-PCR was conducted using an Eco Real-Time PCR system (Illumina, SD, USA), as previously described [11]: 10 min of pre-incubation at 95 °C; followed by 40 cycles of 15 s at  95 °C and 1 min at 60 °C with an AcSir2-specific primer (sense: 5′-CAC CTA CGA CCT CCA TCC GA-3′; antisense: 5′-CTT CTT CCA CTG GAC GGT GAC-3′). Relative amounts were calculated and normalized with respect to the level of *Acanthamoeba* actin as an internal standard (GenBank: CAA23399) [44] (sense primer 5′-AGG TCA TCA CCA TCG GTA ACG-3′ and antisense primer 5′-TCG CAC TTC ATG ATC GAG TTG-3′). The amino acid sequences were aligned using ClustalW and a phylogenetic tree was constructed using Geneious prime (Biomatters, http://www.geneious.com). Bootstrap proportions were used to assess the robustness of the tree with 1000 bootstrap replications.

### Overexpression of AcSir2 in *A. castellanii*

The *AcSir2* gene was cloned into a pGAPDH vector upstream of the enhanced green fluorescent protein (EGFP) reporter gene including an *Acanthamoeba* glyceraldehyde 3-phosphate dehydrogenase (GAPDH) promoter [45]. For transfection into *Acanthamoeba*, *A. castellanii* grown to the mid-log phase were washed with PBS and resuspended in 3 ml of PYG culture medium. Twenty-four hours before transfection, 4 × 10^5^ cells were added to each well of a 6-well culture plate in 3 ml of PYG medium and incubated at 25 °C. Four micrograms of the cloned plasmid (pGAPDH-AcSir2-EGFP) was added to the PYG medium (total 100 μl) and mixed with 20 μl of SuperFect transfection reagent (Qiagen). The mixture was then vortexed for 10 s and incubated for 10 min at room temperature to allow for the formation of the transfection complex. The medium was gently aspirated from the 6-well plate, and the amoeba cells were washed once with 3 ml of PBS. After incubating the DNA with transfection reagent, 1 ml of PYG medium was added to a reaction tube containing the transfection complex and mixed *via* pipetting. The mixture was then immediately transferred to the cells on the 6-well plate. The cells were incubated for 2–3 h at 25 °C, the medium removed, and the cells washed once with PBS before being allowed to recover in 3 ml of PYG medium for 24 h at 25 °C. The transfected cells were then transferred to a selection medium containing 50 μg/ml of G418 for 2–3 weeks until cell growth was apparent. The overexpression of AcSir2-EGFP was assessed using qRT-PCR.

### Confocal microscopy and the determination of the SIRT deacetylase activity of AcSir2

The amoebae expressing EGFP-fused AcSir2 were observed using an LSM 5 Exciter scalable confocal microscope (Zeiss, Hamburg, Germany). EGFP- or DAPI (4′,6-diamidino-2-phenylindole)-mediated fluorescence was performed using band-pass filters at the excitation and emission wavelengths of 500–530 and 360–460 nm, respectively. Images were acquired and analyzed using a Zeiss LSM Image Examiner. SIRT deacetylase activity was measured with a Universal SIRT Activity Colorimetric Assay Kit (Abcam, Cambridge, MA, USA) according to the manufacturer’s instructions. Briefly, nuclear extracts of the vector control and AcSir2-overexpressing trophozoites were prepared using a Nuclear Extract Kit (Thermo Fisher Scientific, Rockford, IL, USA) according to the manufacturer’s recommendations. Nuclear extracts from each sample were incubated with the deacetylase substrate for 1 h at 37 °C and then incubated sequentially with capture and detection antibodies, followed by the colorimetric reaction. Absorbance at 450 nm was measured with a reference absorbance at 655 nm using a microplate reader (Molecular Devices, Sunnydale, CA, USA).

### Transmission electron microscopy (TEM)

*Acanthamoeba castellanii* trophozoites and cysts overexpressing EGFP and AcSir2-EGFP, respectively, were prepared for transmission electron microscopy (TEM) with or without the sirtuin inhibitor salermide as previously described, with slight modifications [11]. Briefly, suspensions of the trophozoites and cysts were centrifuged, and the obtained sediment washed 3 times in cold 1× PBS. The cells were prefixed using 2.5% glutaraldehyde in 0.1 M phosphate buffer (pH 7.4) for 3 h, rinsed with 0.1 M phosphate buffer, post-fixed with 1% osmium tetroxide for 2 h, and rinsed twice with 0.1 M phosphate buffer. The fixed cells were then dehydrated using an ethyl alcohol gradient (50, 70, 80, 95 and 100%), treated twice with propylene oxide resin (1:1) for 20 min, and incubated in propylene oxide resin (1:1) overnight under continuous rotation. The cells were then embedded in epoxy resin (Embed-812; Electron Microscopy Sciences, PA, USA) and incubated at 37 °C for 12 h, 45 °C for 12 h, and 60 °C for 24 h. Ultrathin sections were taken using a Reichert-Jung ultramicrotome and stained with uranyl acetate and lead citrate. The sections were observed using a transmission electron microscope (Hitachi H-7000, Tokyo, Japan).

### Flow cytometry

DNA content and cell size were determined using flow cytometry (FACS Calibur; Becton Dickinson, Franklin Lakes, NJ, USA) and Cell Quest software. For the trophozoite cell size experiments, cells transfected with either the pGAPDH-EGFP or pGAPDH-AcSir2-EGFP plasmid and selected using G418 sulfate (50 μg/ml; Calbiochem, Darmstadt, Germany) were harvested for flow cytometry. To analyze the population of transfected cells, 10,000 EGFP-positive single cells were collected. The mean forward scatter height (FSC-H), which is correlated with cell size, and the optical refraction index of the outer membrane of the cell were determined as a measure of relative cell size [46]. To determine the DNA content, the cells were fixed with 3.7% formaldehyde, permeabilized with ethanol (at a final concentration of 70%) overnight at 4 °C, and then incubated in propidium iodide/RNase A solution (10 μg/ml propidium iodide containing 250 μg/ml RNase A) for 15 min at 37 °C, followed by FACS analysis. FL2-A values correlate with the DNA content.

### Proliferation and encystation assays

To determine the effect of the sirtuin inhibitor salermide (N-{3-[(2-hydroxy-1-naphthalenylmethylene)-amino]-phenyl}-2-phenyl-propionamide; Sigma-Aldrich, MO, USA) on the proliferation and encystation of *A. castellanii*, 3 × 10^5^ cells/well of *A. castellanii* trophozoites were seeded into 6-well plates containing 3 ml of encystment medium per well. Subsequently, the trophozoites were treated with the inhibitor and incubated at 25 °C for 24 and 48 h. The salermide was dissolved in DMSO and stored at − 20 °C until use. The same amount of DMSO was used in the final solutions of the test compound as a control. After incubation, the cells were stained with trypan blue and observed under a microscope. For the encystation suppression assays, encystment was induced and the change from cells to cysts was observed under a light microscope. The encystation ratio was calculated by counting the cysts with a hemocytometer under a light microscope after treating the cells with 0.5% sarkosyl and 0.4% trypan blue [47].

### Statistical analysis

All statistical analyses were conducted using analysis of variance (ANOVA) and Student’s t-tests with GraphPad Prism 8.2 software (GraphPad, San Diego, CA, USA). Significant group differences were evaluated with Tukey’s *post-hoc* multiple comparison tests or Bonferroni correction. *P* < 0.01 (**) was applied to consider significant differences from the control.

## Results

### Identification of the *A. castellanii* silent information regulator 2-like protein

In a previous study, we examined microarray data to identify genes that were differentially expressed in the trophozoite and cyst stages in *A. castellanii* and found a cDNA fragment that exhibited sequence homology with SIR2-like proteins [21]. Because a gene for SIR2-like proteins had not previously been found in *Acanthamoeba* spp., we searched for other SIR2-like proteins by data mining the *A. castellanii* genome database and found four homologues containing the conserved domain of the SIR2 family (pfam02146) but differing in their sequence and length (AcSir2a-d; see Additional file 1: Table S1). Of the four homologues, the deduced amino acid sequence for AcSir2b fully matched that of the cDNA fragment observed in our previous microarray analysis.

To determine whether the four SIR2 homologues were expressed in *Acanthamoeba*, their expression was assessed using qRT-PCR with cDNA from the trophozoites and cysts. Of the four SIR2 homologues, only AcSir2b was expressed in the trophozoites and cysts (Additional file 1: Figure S1). Using RT-PCR and sequence comparisons of the genome and cDNA sequences, the full-length sequence for AcSir2b was determined. AcSir2b was designated as *A. castellanii* Sir2-like protein (*AcSir2*, GenBank: XP_004358245). The *AcSir2* gene contains a 1611-bp open reading frame, encoding a 536-amino acid-long protein with a predicted molecular weight of 60.3 kDa. The primary structure of AcSir2 contains a conserved core domain of the SIR2 family of proteins (residues 24–313) including GAGISXXXGIPXXR, PXXXH, TQNID, HG, and two sets of CXXC that may be zinc finger domains (Additional file 1: Figure S2) [48, 49]. The GAWTK motif, which is similar to the conserved GVWTL motif in class-IV sirtuins, was also found in AcSir2. Interestingly, AcSir2 exhibits one structural difference from SIR2 family proteins in other organisms. The YEATS family domain (pfam03366) [50], which recognizes and binds acetylated lysine, is followed by the SIR2 conserved domain of AcSir2 (residues 443–524). The deduced amino acid sequence of AcSir2 exhibited similarities to SIR2 family proteins in other organisms, including *Naegleria gruberi* (21.9% sequence identity) [51], the sirtuin 6 S homolog in *Xenopus laevis* (22.3% sequence identity), NAD-dependent deacetylase sirtuin-7 in *Chrysochromulina* spp. (21.5% sequence identity), and the SIR2 family transcriptional regulator in *Tetrahymena thermophila* (21.6% sequence identity) (Additional file 1: Figure S2). AcSir2 also exhibited a 24% and 20% amino acid sequence identity with human SIRT6 and SIRT7, respectively (Additional file 1: Table S2).

**Fig. 1:**
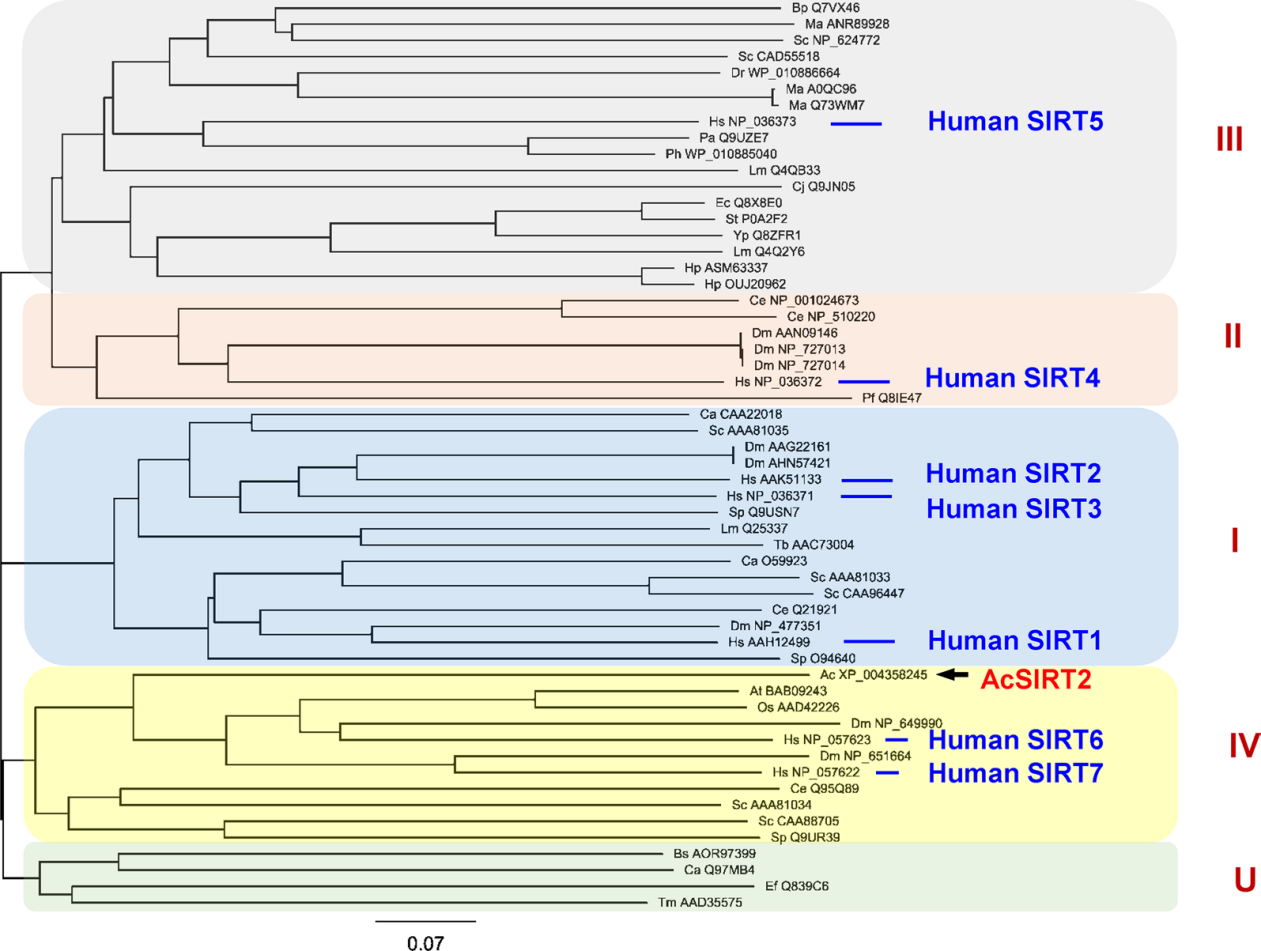
Phylogenetic relationship between AcSir2 and the Sir family in other species. The sequences used in this study are listed in Additional file 1: Table S3

Phylogenetic analysis of the conserved deacetylase domains of AcSir2 in comparison with other SIR2 family proteins from various organisms (Additional file 1: Table S3) revealed that AcSir2 clustered in a clade that is closely related to class IV sirtuins including human SIRT6 and SIRT7 (GenBank: NP_057623 and NP_057622, respectively) and fruit fly sirtuin 6 and sirtuin 7 (GenBank: NP_649990 and NP_651664, respectively) (Fig. 1).

### AcSir2 is a functional SIRT deacetylase in *A. castellanii*

To determine whether AcSir2 was functional, we investigated SIRT deacetylase activity in AcSir2-overexpressing *Acanthamoeba*. AcSir2 was cloned into the GAPDH promoter of the *Acanthamoeba* expression vector (pGAPDH-EGFP) in order to continuously express AcSir2 using C-terminal EGFP fusion (pGAPDH-AcSir2-EGFP). Transfected trophozoites were incubated separately in either PYG medium or encystation medium for 72 h and examined the transcriptional changes of AcSir2 using qRT-PCR. It was subsequently found that the transcriptional levels of the pGAPDH-AcSir2-EGFP transfectants were approximately 250- and 134-fold higher in the trophozoites and cysts compared with the vector control, respectively (*F*_(1, 4)_ = 1186.3, *P* < 0.0001 and *F*_(1,__4)_ = 1017.5, *P* < 0.0001) (Additional file 1: Figure S3).

**Fig. 2:**
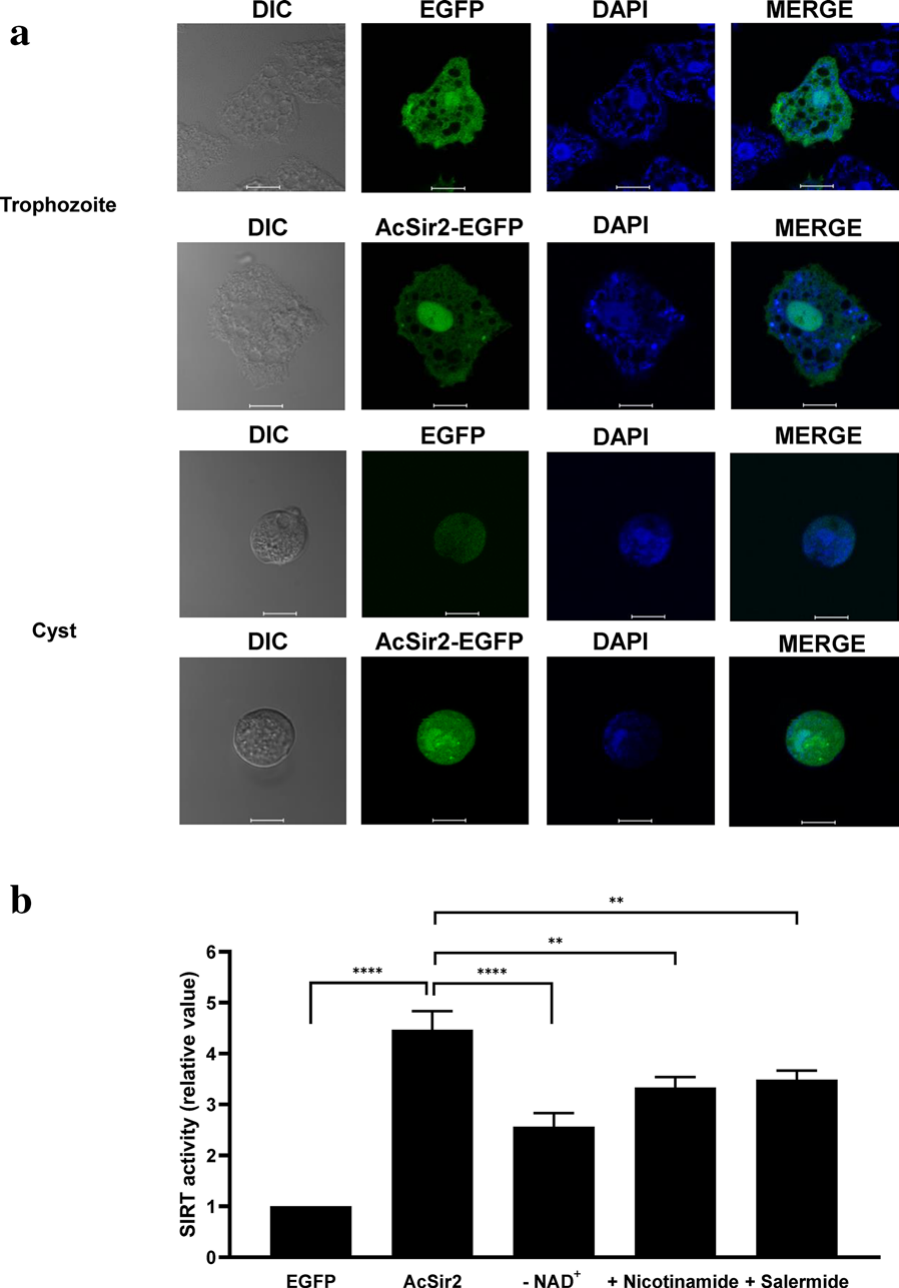
Cellular localization and SIRT deacetylase activity of AcSir2. **a** Intracellular localization of AcSir2 trophozoites (upper panel) transfected with pGAPDH-EGFP and pGAPDH-AcSir2-EGFP (green). Trophozoites were transferred into medium to induce encystation, incubated for 24 h (lower panel), and examined under a fluorescence microscope. Nuclei and mitochondria were visualized using DAPI staining (blue), and resulting merged images are presented. **b** SIRT deacetylase activity of AcSir2 in the nuclear extracts of the vector control (EGFP) and AcSir2-overexpressing trophozoites. Colorimetric assays were conducted with nuclear extracts following treatment with nicotinamide (500 μM) or salermide (500 μM). The data represent the mean ± standard deviation (SD). Significant group differences were evaluated with Tukey’s *post-hoc* multiple comparison tests. **P < 0.01 ***P < 0.001 and ****P < 0.0001. *Scale-bars*: 10 µm

Before determining SIRT deacetylase activity, we examined the intracellular localization of AcSir2 because SIRT2 family proteins are known to differ in their subcellular location. Under a fluorescence microscope after staining with DAPI to label the nucleus, the AcSir2-EGFP fusion protein was mainly found in the nucleus of both the trophozoites and cysts, with lower quantities in the cytoplasm (Fig. 2a). SIRT deacetylase activity was thus measured in the nuclear extracts of AcSir2-overexpressing cells and the vector control. As shown in Fig. 2b, the SIRT deacetylase activity in AcSir2-overexpressing trophozoites was 4 times higher than the vector control (0.1185 ± 0.0033 OD/min/mg *vs* 0.0265 ± 0.0012 OD/min/mg, Tukey’s HSD, *P* < 0.0001). This increase was reduced in the absence of the cofactor NAD^+^ (0.0678 ± 0.0032 OD/min/mg, Tukey’s HSD, *P* < 0.0001) or in the presence of the sirtuin inhibitors nicotinamide (500 μM, 0.0883 ± 0.0060 OD/min/mg, Tukey’s HSD, *P* = 0.0011) and salermide (500 μM, 0.0924 ± 0.0064 OD/min/mg, Tukey’s HSD, *P* = 0.0033). Because SIRT deacetylase activity was higher following the overexpression of AcSir2, it can be concluded that AcSir2 is functional SIRT deacetylase.

### AcSir2 overexpression leads to trophozoite proliferation

**Fig. 3:**
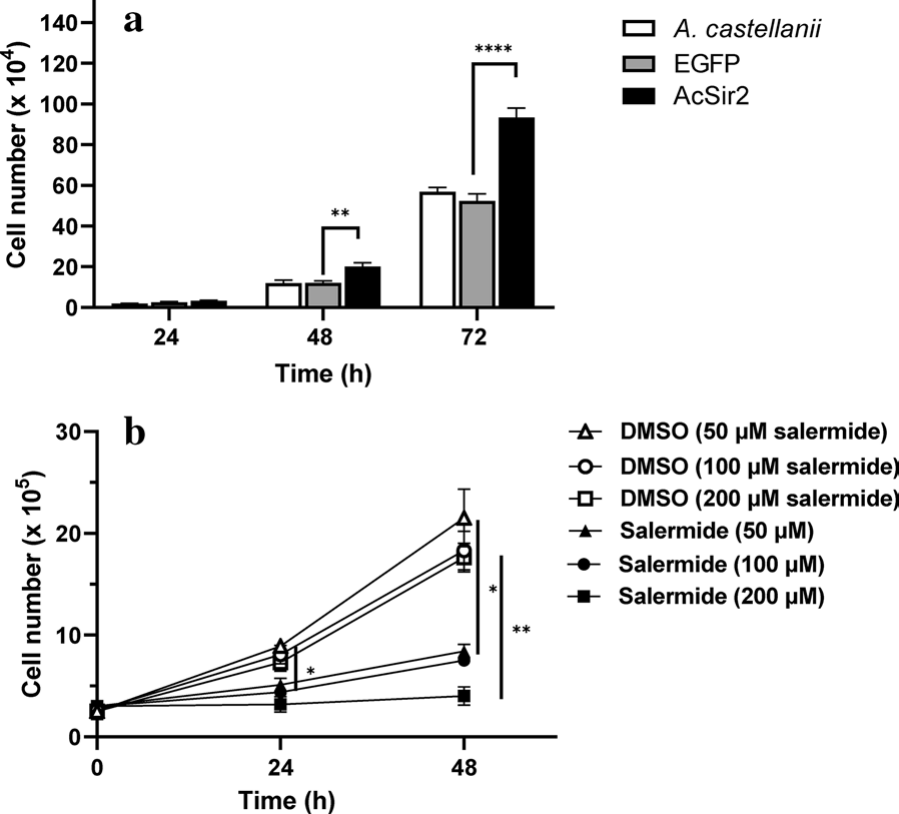
Effects of AcSir2 overexpression on the growth of *A. castellanii* trophozoites. **a** The total cell number of wild-type *A. castellanii* and cells transfected with pGAPDH-EGFP only and pGAPDH-AcSir2-EGFP plasmids 24, 48 and 72 h after incubation in PYG culture medium. The data represent the mean cell number ± standard deviation (SD) (**P < 0.001). **b** Effects of salermide on the proliferation of *A. castellanii* trophozoites. Trophozoites were incubated with various concentrations of salermide (a sirtuin inhibitor) or DMSO (a solvent control). The data represent the mean cell number 0, 24 and 48 h after incubation for each salermide or DMSO concentration. Significant group differences were evaluated with Tukey’s *post-hoc* multiple comparison tests. (**P < 0.01, ***P < 0.001 and ****P < 0.0001

We observed that AcSir2-overexpressing trophozoites exhibited more rapid growth and larger cell sizes than those in the control. To further examine the effect of AcSir2 overexpression on the growth of trophozoites, non-transfected (i.e. wild-type), EGFP-, and AcSir2-overexpressing cells were seeded at equal densities and the number of cells assessed after 24, 48 and 72 h. As shown in Fig. 3a, the cell density of the AcSir2-overexpressing trophozoites was higher, showing a 1.63- and 1.67-fold (Tukey’s HSD, *P* < 0.0001) increase after 72 h compared to the wild-type and EGFP-overexpressing cells, respectively. In order to confirm that the increase in cell number was due to the overexpression of AcSir2, the cells were treated with various concentrations of the sirtuin inhibitor salermide and the number of cells assessed 24 and 48 h after treatment. As shown in Fig. 3b, after 24 h, the number of salermide-treated cells increased by 36.7, 25.5 and 0.01%, compared to those of DMSO-treated cells at 50, 100 and 200 μM salermide, respectively (*t*_(4)_ = 5.97, *P* = 0.004). After 48 h of treatment with 200 μM salermide, the difference in cell number became more significant between DMSO- and salermide-treated cells (Tukey’s HSD, *P* = 0.0092), indicating that salermide inhibited the proliferation of *A. castellanii* trophozoites in a dose- and time-dependent manner and that the higher cell density of *A. castellanii* trophozoites was due to AcSir2 overexpression.

**Fig. 4:**
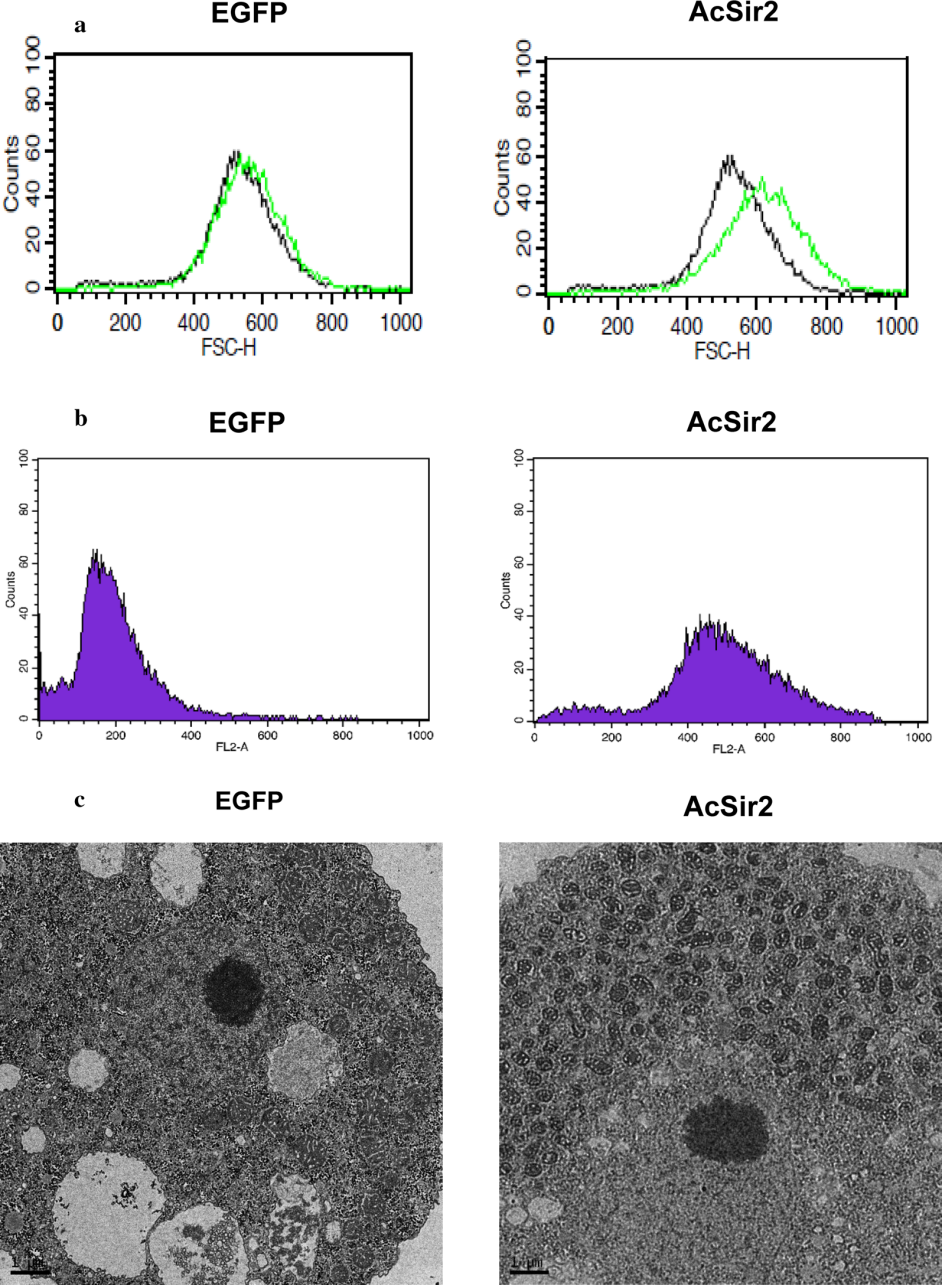
Effects of AcSir2 overexpression on *Acanthamoeba* trophozoites. **a** Wild-type (*A. castellanii*, black) and trophozoites overexpressing either EGFP (left, green) or AcSir2 (right, green) were analyzed on a flow cytometer for changes in cell size using the mean FSC-H. **b** The DNA content of the trophozoites overexpressing either EGFP (left) or AcSir2 (right) was determined after PI staining using flow cytometry. The data represent one of three experiments with similar results. **c** Ultrastructural changes in AcSir2-overexpressing cells. Trophozoites transfected with the vector control (pGAPDH-EGFP) (left) and pGAPDH-AcSir2-EGFP (right) were examined using transmission electron microscopy (TEM). *Scale-bars*: **c**, 1 μm

Because AcSir2-overexpressing cells exhibited a difference in cell size under a light microscope, we analyzed EGFP and pGAPDH-AcSir2-EGFP transfectants using flow cytometry to quantitatively determine the cell size based on the measurement of mean FSC-H, a parameter used routinely to measure the size of cells [52]. The overexpression of AcSir2 shifted the mean FSC-H histogram rightward (mean FSC-H = 609.19) compared with the vector control (mean FSC-H = 527.17) (Fig. 4a), indicating that the size of the AcSir2-overexpressing cells was larger by 116%. The EGFP- and AcSir2-overexpressing trophozoites were then stained with propidium iodide (PI) and their DNA content examined using flow cytometry. As shown in Fig. 4b, the FL2 values for the AcSir2-overexpressing cells (454.74 ± 30.63) exhibited a large rightward shift compared with the control (159.77 ± 48.91), meaning that the overexpression of AcSir2 in trophozoites increased the DNA content by 284.6%. Subsequently, the ultrastructural changes in AcSir2-overexpressing cells were examined using TEM. As shown in Fig. 4c, a higher electron density and an increase in the number of mitochondria were observed in AcSir2-overexpressing trophozoites (right) compared with the vector control (left), showing that the overexpression of AcSir2 increased the intracellular content of mitochondria.

### AcSir2 is highly expressed during the encystation of *A. castellanii* and its overexpression accelerates the encystation process

**Fig. 5:**
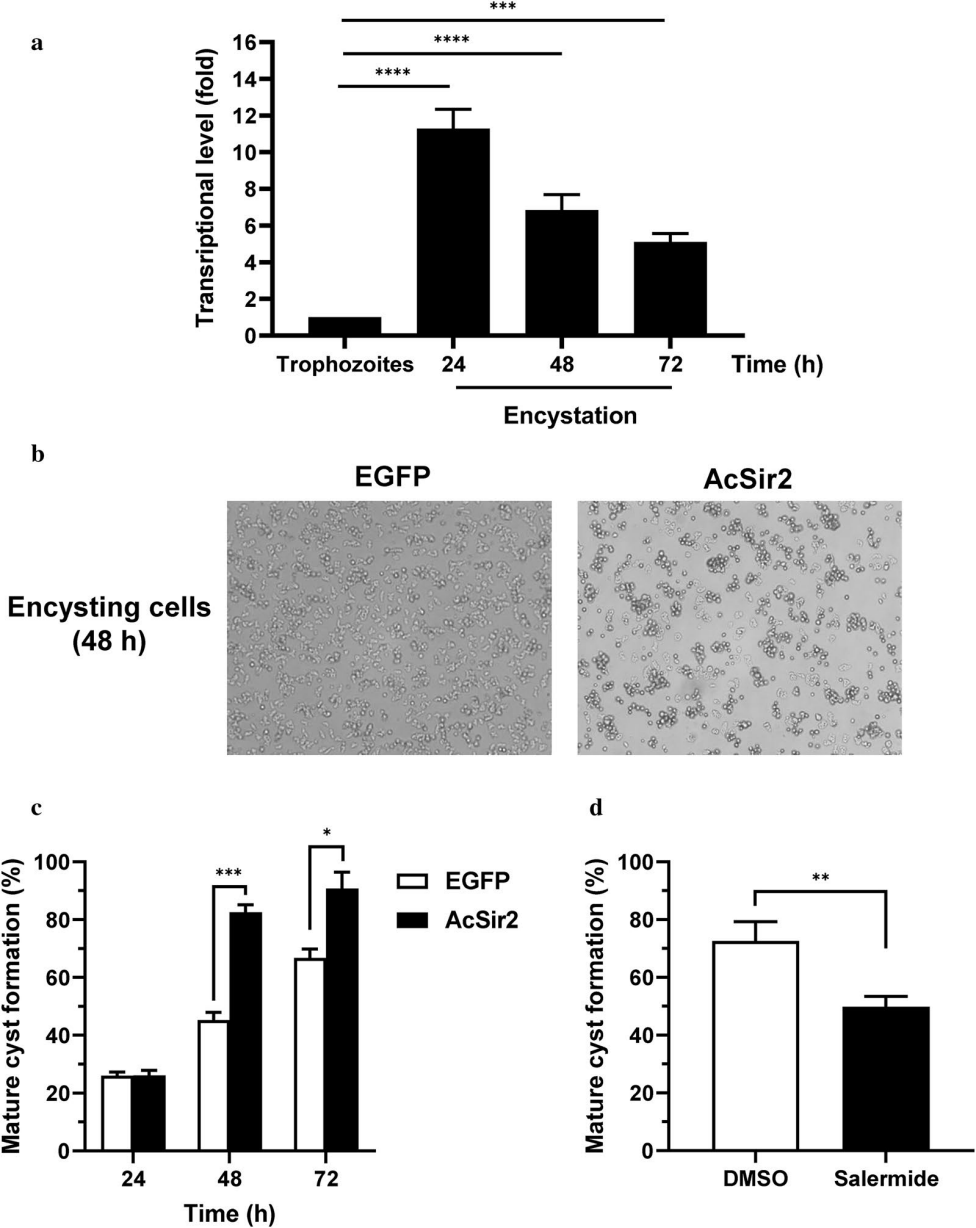
Transcriptional changes in AcSir2 during encystation and the effects of AcSir2 overexpression on the encystation of *A. castellanii*. **a** Changes in the expression of AcSir2 during the encystation of *A. castellanii*. Trophozoites and encysting cells 24, 48 and 72 h after the induction of encystation were examined in terms of AcSir2 transcriptional changes using qRT-PCR. *Acanthamoeba* actin was used as an internal control. **b**, **c** Effects of AcSir2 overexpression on the encystation of *A. castellanii*. Vector control (left) and AcSir2-overexpressing trophozoites (right) were transferred into encystation medium, incubated for 48 h, and examined directly under a microscope (**b**). Encysting cells overexpressing EGFP and AcSir2 at 24, 48 and 72 h after treatment with 0.5% sarkosyl for 30 min and staining with 0.4% trypan blue, with the number of mature cysts counted under a microscope (**c**). **d** Effects of salermide on the encystation of *A. castellanii* trophozoites. *A. castellanii* trophozoites were transferred into encystation medium containing 100 μM of salermide and incubated for 72 h. The mature cysts were counted under a microscope, followed by treatment with sarkosyl. DMSO was used as a solvent control. The percentage of mature cysts is calculated as the mean number of cells remaining after sarkosyl treatment compared with the total number of cells. The data represent the mean ± standard deviation (SD) of three separate experiments. **P < 0.01, ***P < 0.001 and ****P < 0.0001

Changes in the expression of AcSir2 in both trophozoites and encysting cells were determined using qRT-PCR. As shown in Fig. 5a, the transcriptional levels of AcSir2 increased approximately 11-fold at 24 h post-encystation (*P* < 0.0001); these levels gradually decreased at 48 and 72 h post-encystation, but remained higher than those in trophozoites. Under a light microscope, we observed that a larger number of cysts formed in the AcSir2-overexpressing cells than in the control cells after the induction of encystation (Fig. 5b). To ascertain whether greater AcSir2 expression affects the encystation of *A. castellanii*, vector control and AcSir2-overexpressing trophozoites were transferred to encystation medium to induce encystation and incubated for 24, 48 and 72 h, and mature cysts were counted under a microscope after treatment with sarkosyl. As shown in Fig. 5c, both the vector control and AcSir2-overexpressing cells encysted gradually over time, though the encystation rate was significantly higher in the latter group. In particular, at 48 h post-encystation, 45.3% of the vector control cells had transformed into mature cysts, compared to 82.5% of the AcSir2-overexpressing cells (*post-hoc* pairwise tests with Bonferroni correction; *P *= 0.0002).

We then looked to verify whether the more rapid encystation was specifically due to the overexpression of AcSir2. The encysting trophozoites were treated with various concentrations of salermide and the encystation rate determined. As shown in Fig. 5d, 49.8% of cells treated with 100 μM of salermide transformed into cysts at 72 h post-encystation, which was significantly lower than DMSO-treated control cells (*t*_(4)_ = 5.119, *P* = 0.0069). This indicates that sirtuin inhibitors suppress the encystation of *A. castellanii* and that encystation is accelerated by the overexpression of AcSir2.

### Ultrastructural changes occur following treatment with a sirtuin inhibitor in encysting *A. castellanii*

**Fig. 6:**
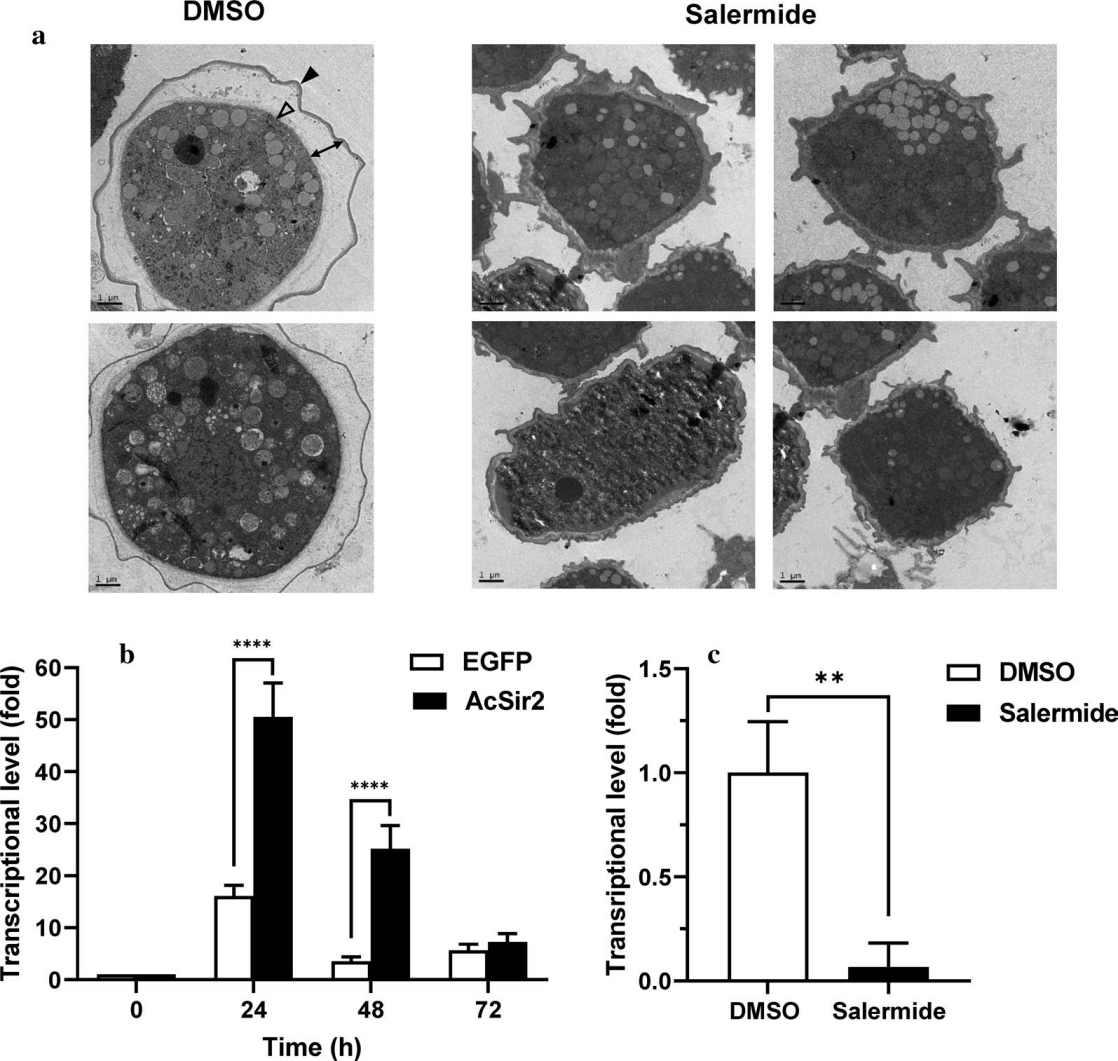
Inhibition of the encystation of *A. castellanii* with salermide treatment. **a** Ultrastructural changes following treatment with salermide in the encysting of *A. castellanii*. Encystation was induced by transferring cells into encystation medium containing DMSO (left) or 100 μM salermide for 24 h (right). The same amount of DMSO (as a solvent control) was also added to the encystation medium. The black arrowhead, empty arrowhead and black double-headed arrows indicate the exocyst wall, endocyst wall and intercyst space, respectively. **b** Transcriptional changes in cellulose synthase in encysting AcSir2-overexpressing cells. AcSir2-overexpressing trophozoites (0 h) and encysting cells at 24, 48 and 72 h after the induction of encystation were examined for transcriptional changes in cellulose synthase using qRT-PCR. The transcriptional levels of cellulose synthase were normalized to that of *Acanthamoeba* actin. **c** Effects of salermide on transcription levels in cellulose synthase during the encystation process. Trophozoites were transferred into encystation medium containing 100 μM of salermide, incubated for 24 h, and examined for transcriptional changes in cellulose synthase using qRT-PCR. The transcriptional levels of cellulose synthase in the salermide-treated cells were compared to those of DMSO-treated cells. **P < 0.01, ***P < 0.001 and ****P < 0.0001. *Scale-bar*: **a**, 1 μm

We then examined the morphological changes in encysting cells treated with a sirtuin inhibitor. Differences between DMSO- and salermide-treated (100 μM) cells were examined using TEM. The DMSO-treated cells transformed into mature double-walled cysts with a laminar fibrous ectocyst wall (Fig. 6a, left, filled arrowheads) and an endocyst wall composed of fine fibrils in a granular matrix (Fig. 6a, left, open arrowheads), which is a morphology typical of *Acanthamoeba* cysts [5]. Vesicular structures were also detected, and the partial degradation of organelles and intracellular compartments within these structures was observed at 24 h post-encystation (Fig. 6a, left). In comparison, when *A. castellanii* cells were transferred into encystation medium containing 100 μM salermide for 24 h, the ectocyst wall was observed to be deeply wrinkled and thicker than that of the DMSO-treated cells, which exhibited thorn-like spines protruding from the cytoplasm (Fig. 6a, right). The endocyst wall and intercyst space separating the ectocyst and endocyst walls (Fig. 6a, left, double-headed arrow), which are typically observed in encysting *Acanthamoeba*, were noticeably absent in the salermide-treated encysting cells (Fig. 6a, right), suggesting that salermide significantly impairs the formation of the cyst wall during the encystation process.

### The transcriptional regulation of *A. castellanii* encystation-mediating proteins

To investigate the cause of the more rapid encystation in AcSir2-overexpressing cells and the impaired formation of the cyst wall in salermide-treated *A. castellanii* cells, we first examined the transcriptional levels of cellulose synthase, which is known to be upregulated in encysting cells [17]. The transcriptional levels of cellulose synthase in AcSir2-overexpressing cells were 3.1- and 7.2-fold higher at 24 and 48 h after the induction of encystation, respectively, compared with vector control cells (Tukey’s HSD, *P* < 0.0001) (Fig. 6b), while the transcription of cellulose synthase was completely abolished in salermide-treated cells (*t*_(4)_ = 5.954, *P* = 0.004) (Fig. 6c). These results show that sirtuin regulates the transcriptional levels of cellulose synthase during encystation.

## Discussion

In mammals, there are seven sirtuin isoforms (Sirt1–7), and sirtuins have been classified into five major classes (I, II, III, IV and U) that differ in their intracellular distribution [27, 49, 53, 54]. Phylogenetic analysis comparing the conserved catalytic domain of AcSir2 with the sirtuins reported from various organisms revealed that AcSir2 is a class IV sirtuin, as are SIRT6 and SIRT7, which are found in humans, fruit flies and plants (Fig. 1). Consistent with initial reports on the nuclear localization of human SIRT6 and SIRT7 [25], EGFP-tagged AcSir2 was predominantly localized in the nucleus. In protozoan parasites, class-IV sirtuins have previously only been found in the phylum Apicomplexa (*Plasmodium*, *Toxoplasma*, *Neospora*, *Theileria*, *Babesia* and *Eimeria*), while class I, II, III, and U sirtuins have been reported in the phylum Sarcomastigophora, such as *Trypanosoma*, *Leishmania*, *Giardia* and *Trichomonas* (see [33] for a review). Thus, AcSir2 is the first class IV sirtuin to be reported in Sarcomastigophora. Interestingly, AcSir2 has a YEATS domain, which is named for the five proteins that contain this domain (Yaf9, ENL, Af9, Taf14 and Sas5) and which recognizes and binds acetylated lysine. The YEATS domain functions as a docking site for reader proteins and is evolutionarily conserved in organisms ranging from yeast to humans. This domain is observed in a variety of nuclear complexes with molecular functions ranging from histone modification to transcription regulation [55, 56]. However, no YEATS domain has been observed in SIR2-like proteins in other organisms. The AcSir2 protein contains the YEATS domain followed by a Sir2 catalytic domain that functions as an NAD^+^-dependent deacetylase. This structural characteristic may contribute to more effective SIRT deacetylase activity. This structure is also likely to be a primitive form of NAD^+^-dependent deacetylase, supposing the evolutionary divergence into sirtuin family proteins with the Sirt2 domain and transcription-regulating proteins with only the YEATS domain. Further research using YEATS domain deletion mutants of AcSir2 is required to examine SIRT deacetylase activity and compare the effects on encystation with wild-type AcSir2.

Mammalian SIRT1–3 have strong deacetylase activity, while SIRT4–7 are known to have weak or non-detectable deacetylase activity [57]. In the present study, nuclear extracts of wild-type *A. castellanii* trophozoites exhibited non-detectable deacetylase activity (Fig. 2b). Thus, we examined the deacetylase activity in AcSir2-overexpressing cells. Incubation of the nuclear extracts of AcSir2-overexpressing cells with an acetylated substrate in the presence of NAD+ cofactor exhibited deacetylase activity (Fig. 2b). As shown in Fig. 2b, the greater SIRT deacetylase activity due to the overexpression of AcSir2 was weakened in the presence of nicotinamide, one of the products of the deacetylation reaction [58], and salermide, a sirtuin inhibitor, verifying that AcSir2 has SIRT deacetylase activity.

In this study, *A. castellanii* AcSir2-overexpressing trophozoites had greater cell density compared with the vector control (Fig. 3a). In salermide-treated AcSir2 overexpressing cells, proliferation of trophozoites was not significantly inhibited due to the residual SIRT deacetylase activity (Fig. 2b and Additional file 1: Figure S4). Salermide suppressed the growth of wild type *A. castellanii* trophozoites in a dose- and time-dependent manner (Fig. 3b). These results indicate that the higher cell density was a result of the overexpression of AcSir2. In yeast, worms and flies, Sir2 homologs positively regulate longevity [59–63], while Ford et al. [64] suggested that SIRT7 is a nuclear protein that positively regulates RNA polymerase I and is required for cell viability in mammals. In actively proliferating trophozoites, the higher cell density could be attributed to either more rapid cell division or increased longevity; which of these mechanisms is dominant has not yet been clarified for AcSir2-overexpressing cells. Also, the used plasmid vectors were reported to be maintained episomally [45]; we open the possibility that cell death could be delayed due to deleterious mutations or homologous recombination by transfected plasmids.

The AcSir2-overexpressing cells were also observed to be larger in size, with a rightward shift observed in the FSC-H histograms when compared with the control (Fig. 4a). To examine the reason for this increase in cell size, DNA staining using PI revealed higher DNA content in AcSir2-overexpressing cells. In the TEM analysis, ultrastructural changes in AcSir2-overexpressing cells indicates that this increase in DNA content was due to an increase in the number of mitochondria (Fig. 4c). Thus, it is likely that the increase in cell size of AcSir2-overexpressing cells was caused by the increase in mitochondrial DNA. In mammals, SIRT1, which is primarily nuclear, has been implicated in mitochondrial biogenesis *via* the deacetylation of target proteins such as hypoxia-inducible factor 1α (HIF-1α) [65] and peroxisome proliferator-activated receptor gamma coactivator-1 (PGC-1) [66]. When searching the *Acanthamoeba* genome, we only found a homologue of HIF-1α, thus further studies on the deacetylation of target proteins by AcSir2 are required to determine its importance in mitochondrial biogenesis.

Sir2-like proteins, which are NAD^+^-dependent deacetylases, sense intracellular levels of NAD^+^, which are affected by caloric uptake [67, 68], and communicate the nutrient status of the external environment to the different intracellular compartments within the cell. The encystation of *Acanthamoeba* occurs to ensure its survival and transmission in a variety of adverse environmental conditions, including the depletion of nutrients. In the present study, although it remains unknown whether AcSir2 acts as a nutrient and metabolic sensor in *A. castellanii*, the transcription of AcSir2 was upregulated during encystation (Fig. 5a). The overexpression of AcSir2 following transfection with an AcSir2-cloned *A. castellanii* expression vector accelerated the encystation process, converting cells into mature cysts more efficiently (Fig. 5b, c), while the encystation of *A. castellanii* was suppressed by salermide treatment (Fig. 5d). Previous research has shown that, in *S. cerevisiae*, SIR2 levels increase during calorie restriction [69, 70], and sirtuin overexpression is known to extend the lifespan by silencing HML and HMR loci and inhibiting the formation of extrachromosomal rDNA circles [71, 72]. Of the *T. cruzi* sirtuins, TcSir2rp3 not only increases parasite invasion and multiplication, but the overexpression of TcSir2rp3 enhances parasite growth and increases the transformation of epimastigotes to metacyclic trypomastigotes due to starvation [36]. In addition, as in *A. castellanii*, salermide reduced the growth and differentiation of *T. cruzi*. Despite this mass of evidence for the importance of sirtuins in the encystation process, further research is needed to determine what changes occur at the intracellular level during encystation in AcSir2-overexpressed cells. In addition, it has been reported that the *Acanthamoeba* strain used in this study loses its potential for encystment by long-term culture under axenic condition [73]. Therefore, the effects of salermide treatments on encystation should be evaluated in fresh isolates or other strains of *Acanthamoeba*.

*Acanthamoeba* cysts have an ectocyst wall (the outer cyst wall) consisting of acid-insoluble protein-containing materials and an endocyst wall (the inner cyst wall), which is partially composed of cellulose [3, 7, 8]. Recent studies have shown that cellulose fibrils exist in both cyst walls, and cellulose synthesis has been suggested as an important target for the treatment of *Acanthamoeba* infections [74]. Previous research on cellulose synthesis inhibitors and the knockdown of *Acanthamoeba* cellulose synthase has found that cellulose is essential to the formation of cyst walls during encystation [17, 75]. It is known that the transcriptional levels of cellulose synthase are upregulated during the encystation process [76]. In the present study, the transcriptional increase in cellulose synthase in AcSir2-overexpressing cells was more significant than in the control (Fig. 6b). However, the transcription of cellulose synthase was completely abolished in salermide-treated cells (Fig. 6c). In TEM analysis, we found that the structure of the ectocyst wall, intercyst space, and endocyst wall was subsequently impaired by salermide treatment. In particular, the endocyst wall, which consists of polysaccharides such as cellulose [4, 77], was absent in salermide-treated encysting cells (Fig. 6a). Thus, it is likely that that the absence of endocysts in the salermide-treated cells was due to the absence of cellulose arising from the lack of cellulose synthase.

## Conclusions

We identified the sirtuin-like protein AcSir2 in *A. castellanii*, which, to the best of our knowledge, is the first class-IV sirtuin reported in the phylum Sarcomastigophora. AcSir2 has functional SIRT deacetylase activity and is localized primarily in the nucleus. AcSir2 overexpression leads to increased cell growth in *A. castellanii* trophozoites and accelerated encystation, and these changes were reverted by salermide treatment. The regulation of AcSir2 expression is found to be important for the growth and encystation of *Acanthamoeba*, thus highlighting its potential worth as a therapeutic target.

## Supplementary information

**Additional file 1: Table S1.** The predicted amino acid sequences of the genes with the conserved domain of SIR2 family proteins following the screening of the *Acanthamoeba* genome database. **Figure S1.** Transcriptional profiles were obtained with qRT-PCR for four SIR2 homologues using cDNAs from *A. castellanii* trophozoites (T) and encysting cells (C) at 24, 48, and 72 h after induction of encystation. **Figure S2.** Comparison of AcSir2 (XP_004358245) with sirtuin-like proteins from other organisms. **Table S2.** Distance matrix of the identity scores (%) resulting from the alignment of AcSir2 with human sirtuin proteins. **Table S3.** GenBank accession numbers and description of Sir2 family proteins in terms of multiple alignment and phylogeny. **Figure S3.** Overexpression of AcSir2 in *A. castellanii* trophozoites and cysts. **Figure S4.** Effects of salermide on proliferation of *A. castellanii* trophozoites (upper), and EGFP- (middle) and AcSir2-EGFP (lower) overexpressing trophozoites.

## Data Availability

The data supporting the conclusions of this article are included within the article and its additional file. The plasmids are available on request.

## Abbreviations

AcSir2: *Acanthamoeba castellanii* sirtuin 2
NAD: nicotinamide nucleotide dinucleotide
EGFP: enhanced green fluorescent protein
RT-PCR: reverse transcription-polymerase chain reaction
qRT-PCR: quantitative real-time PCR
TEM: transmission electron microscopy
PI: propidium iodide
FSC-H: forward scatter height
HIF-1α: hypoxia-inducible factor 1α
PGC-1: peroxisome proliferator-activated receptor Gamma Coactivator-1
