# Supplementary material for: The role of the Acanthamoeba castellanii Sir2-like protein in the growth and encystation of Acanthamoeba
Source: Parasit Vectors. 2020 Jul 22;13:368. doi: 10.1186/s13071-020-04237-5 (PMC7376869; doi:10.1186/s13071-020-04237-5)
Supplement: Supplementary file 1 — Additional file 1: Table S1. The predicted amino acid sequences of the genes with the conserved domain of SIR2 family proteins following the screening of the Acanthamoeba genome database. Figure S1. Transcriptional profiles were obtained with qRT-PCR for four SIR2 homologues using cDNAs from A. castellanii trophozoites (T) and encysting cells (C) at 24, 48, and 72 h after induction of encystation. Figure S2. Comparison of AcSir2 (XP_004358245) with sirtuin-like proteins from other organisms. Table S2. Distance matrix of the identity scores (%) resulting from the alignment of AcSir2 with human sirtuin proteins. Table S3. GenBank accession numbers and description of Sir2 family proteins in terms of multiple alignment and phylogeny. Figure S3. Overexpression of AcSir2 in A. castellanii trophozoites and cysts. Figure S4. Effects of salermide on proliferation of A. castellanii trophozoites (upper), and EGFP- (middle) and AcSir2-EGFP (lower) overexpressing trophozoites. [file 13071_2020_4237_MOESM1_ESM.pdf]

Additional file 1: Table S1. The predicted amino acid sequences of the genes with the conserved domain of SIR2 family proteins following the screening of the *Acanthamoeba* genome database.

Temporary ID	AcSir2a	GenBank accession no.	XP_004353652	No. of Amino acids	582

```
METTSFDEEKTGFYVTPKLDCPHIAQHVRCNASKEALKAVAALGPCETCHDPKENWACLQCGKKFCSRYVAGHMK
EHNTQSGHAITVSYSDFSFWCYECDSYIAHDIFRPLLALLKESKFGDAGTGGRYTAHKTHTQMEEIEEKPEELAA
KIKALAGMIRDSKHCVFFTGAGVSTSAGIPDYRGPEGVWTLKATGGQRKTKAVPMLSALPTVTHMAMVKLHDVDR
MHYLVSQNVDGIHRKSGIHPQRLCELHGNSNLEVCCWCGKEYMRDFDTCHNSAAGSHETGRRCTAPGCGGPLLDT
IINFGENLPKKDLERAYDECDKADLIVCLGSSLTVSPANDLPKRVAKRGGNLVIVNLQRTPLDSLSTLRIHGRTD
EVMKGVMEELGIEVPSFILNRFVRVQHTKQSLTVEALDVDGTPISLFTTVKAQFLPAGKVHKKQTQSVLGRMETS
YVFERTGDEDASEVKIELQFMGHYREPSFRFTYALEKESSDKRFMISFNPEEARWDIKEVESAGAAETEGKTGPE
HWPKMAQDSTHEHPLKLKKAVYNGIYRCNKCMRPGTGWVYHCKPCAFDLHAFCCDKH
```

Temporary ID	AcSir2b	GenBank accession no.	XP_004358245	No. of Amino acids	536

```
MASTVDSTRVREFNEENDPFFQKKLDRLAKMVKQSKYTVFFTGAGVSTSAGVGDYRGPSGAWTKRKIKELELLGA
GRTAEDESELTKLKAEAAREEKKARVKIDMCDAQPTPTHMAMATLIRLGLAHYVVTTNLDGIYRKAGLKDHEQLC
CLHGDIYVERCSGCGYDFERNYHVRQGHTHVHDHKVGTCSRCGSAPPAHYKGTPGDLKMKKGRWGGRMVGTRDTN
CGTKDTHINFGELLDEVDWNEADTHCRRADLCIIAGTSMSLRHITHFPFLARQVVLINLQPTPDDDEATLRIWAK
CDPVFEGLMARLDVPIDPVPVWRPRDAVPLNRIPNFVHPYYKKKAEAIEEMARLREAEADDRRREAAAERLTRQV
DRMEVVDVSSSSEEDDEAEAGEKKNKKKSKKTKTKKEKNEDDEVVEVPKVIEVGNEHAAVPTDRGNNTHQWTMFV
KLPDGRDDARDLAELVDHVTYDLHPTFSPAQVRVTEAPFRVTRLGWGTFSVGVTVQWKKHVGHAPLRCKHTLSFG
TAKASELIDVA
```

Temporary ID	AcSir2c	GenBank accession no.	XP_004367869	No. of Amino acids	388

```
MASTVDSTRVREHSEENEPLFEEKLDRLAKMVRRSRYTVFFTGAGVSTSAGVGDYRGPEGAWTRRKIKQLEALGP
LRSVEDESELRKLKEEAARAEKKAKARVDMCDAQPTPSHMAMATLLRLGLAHYVITTNLDGIFRKAGLQAHEQLC
CLHGDIYVERCTSCDYDFERNYHVRQPEVHVHDHKVGTCARCGSAPPAHYTGTPGNLKMQNGRWGGRMVGTRDRN
CGTKDTHINFGELLDEVDWTEADTHCRRADLCIIAGTSMSLRHITHFPFLARRVVLVNLQPTPDDHKVALRLWAK
CDPVFEGLMARLGLAIDPIPPWRPRDALPLDRLPAYVHPYYKMKAQLLEQMALLREAEAEDRRREQEQQQEEEG
EKEVVGRGYQPMR
```

Temporary ID	AcSir2d	GenBank accession no.	ELR17704	No. of Amino acids	223

```
MSEQKPTNDEQKETTEEVTKEEEEGGLFNGALNLLEQLKGLSVEDMRAMLKAPPQPEKVLSEPTLEAVAEAIREG
KCHKVIVMAGAGISVAAGIPDFRSPGTGLYDNLQVRPCKYNLPHPTAVFELGYFKTNPKPFYTLAKELYPGSFVP
TPAHHLVKLLHDKGVLLRAYTQNIDGLERIAGVPDDKIIEAHGAALSTSTAMSRVGSPERTWECGVLSRQDWN
```

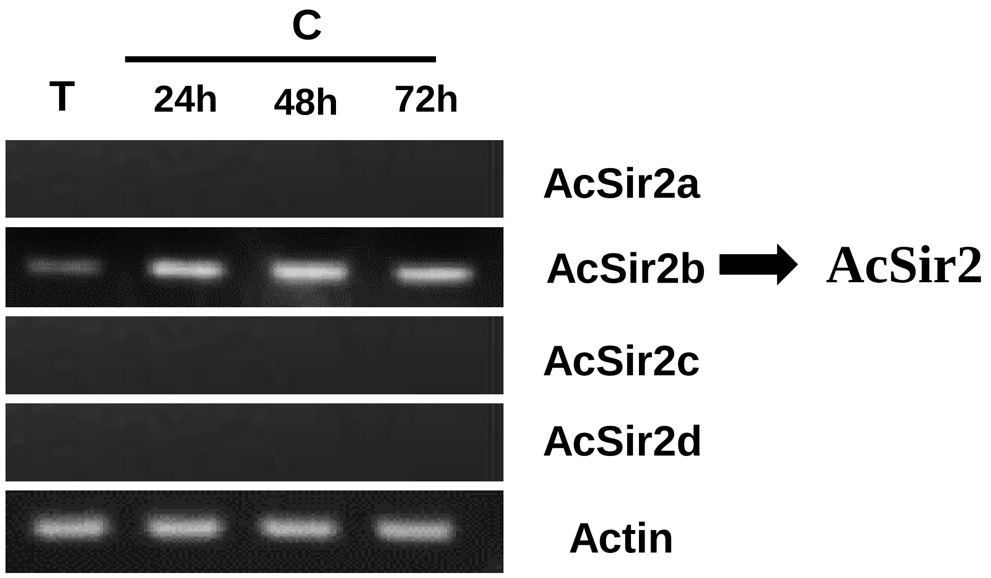

Additional file 1: Figure S1. Transcriptional profiles were obtained with qRT-PCR for four SIR2 homologues using cDNAs from *A. castellanii* trophozoites (T) and encysting cells (C) at 24, 48, and 72 h after induction of encystation. *Acanthamoeba* actin was used as an internal control

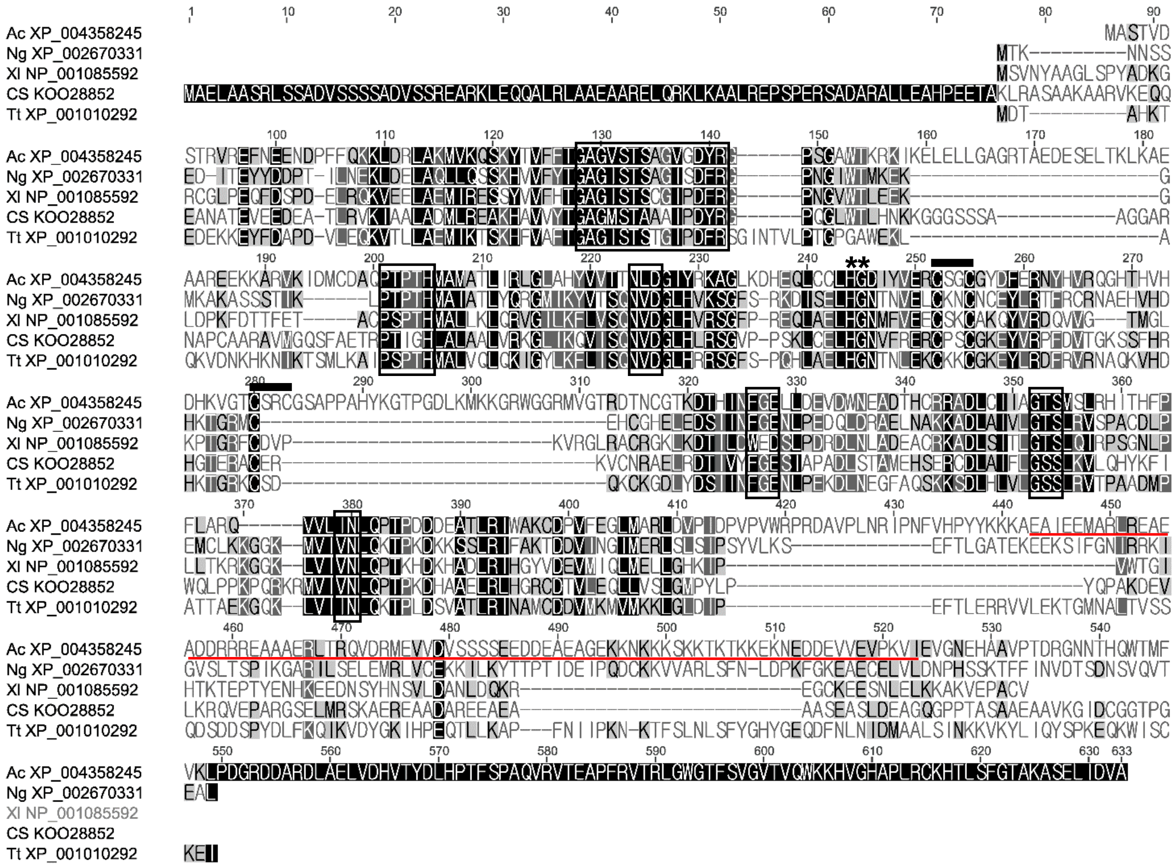

Additional file 1: Figure S2. Comparison of AcSir2 (Ac XP_004358245) with sirtuin-like proteins from other organisms: (A) *Naegleria gruberi* (Ng XP_002670331), *Xenopus laevis* (Xl NP_001091351), *Chrysochromulina* sp. (CS KOO28852), and *Tetrahymena thermophila* (Tt XP_001010292S). The sirtuin homology domains are shaded. The conserved "HG" is marked (**). The putative zinc finger domain motifs previously noted in SIR2-like proteins are designated with overlying bars. The conserved GAGISXXXGIPXXR, PXXXH, TQNID, FGE, GTS, and (I/V)N motifs and the YEATS family domain are indicated with boxes and red lines, respectively.

Additional file 1: Table S2. Distance matrix of the identity scores (%) resulting from the alignment of AcSir2 with human sirtuin proteins. Hs denotes *Homo sapiens*. The GenBank accession number of each gene is given in Additional File 4. The highest identity shared with AcSir2 is indicated by the red-colored box.

	**Hs SIRT1**	**Hs SIRT2**	**Hs SIRT3**	**Hs SIRT4**	**Hs SIRT5**	**Hs SIRT6**	**Hs SIRT7**	**AcSir2**
**Hs SIRT1**		32.5%	30.2%	16.2%	15.8%	13.4%	15.5%	12.0%
**Hs SIRT2**	32.5%		48.7%	18.1%	20.9%	18.3%	19.5%	14.9%
**Hs SIRT3**	30.2%	48.7%		19.7%	22.1%	23.5%	23.5%	15.9%
**Hs SIRT4**	16.2%	18.1%	19.7%		21.6%	19.8%	21.1%	17.7%
**Hs SIRT5**	15.8%	20.9%	22.1%	21.6%		16.7%	19.1%	16.2%
**Hs SIRT6**	13.4%	18.3%	23.5%	19.8%	16.7%		42.1%	23.6%
**Hs SIRT7**	15.5%	19.5%	23.5%	21.1%	19.1%	42.1%		23.6%
**AcSir2**	12.0%	14.9%	15.9%	17.7%	16.2%	23.6%	23.6%	

Additional file 1: Table S3. GenBank accession number and description of Sir2 family proteins for multiple alignment and phylogeny.

Species	Description	Length (aa)	GenBank Accession no.
Acanthamoeba castellanii	transcriptional regulator, Sir2 family	536	XP_004358245
Arabidopsis thaliana	transcription regulator Sir2-like protein	473	BAB09243
Bordetella pertussis	SIR2 homolog	274	Q7VX46
Bacillus subtilis	NAD-dependent protein deacylase	247	AOR97399
Candida albicans	transcription regulatory protein	331	CAA22018
Candida albicans	SIR2	519	O59923
Clostridium acetobutylicum	SIR2 homolog	245	Q97MB4
Caenorhabditis elegans	NAD-dependent protein deacylase sir-2.2	289	NP_001024673
Caenorhabditis elegans	NAD-dependent protein deacylase sir-2.3	287	NP_510220
Caenorhabditis elegans	sir-2.1	607	Q21921
Caenorhabditis elegans	SIR2 homolog 4	292	Q95Q89
Campylobacter jejuni	SIR2 homolog	233	Q9JN05
Drosophila melanogaster	sirtuin 2, isoform A	355	AAG22161
Drosophila melanogaster	sirtuin 4, isoform C	312	AAN09146
Drosophila melanogaster	sirtuin 2, isoform B	355	AHN57421
Drosophila melanogaster	sirtuin 1	823	NP_477351
Drosophila melanogaster	sirtuin 6	325	NP_649990
Drosophila melanogaster	sirtuin 7	771	NP_651664
Drosophila melanogaster	sirtuin 4, isoform A	229	NP_727013
Drosophila melanogaster	sirtuin 4, isoform B	229	NP_727014
Deinococcus radiodurans	NAD-dependent deacylase	246	WP_010886664
Escherichia coli	SIR2 homolog class III	279	Q8X8E0
Enterococcus faecalis	SIR2 homolog	237	Q839C6
Helicobacter pylori	NAD-dependent deacylase	229	ASM63337
Helicobacter pylori	NAD-dependent deacylase	234	OUJ20962
Homo sapiens	SIRT1 protein	555	AAH12499
Homo sapiens	SIRT2	352	AAK51133
Homo sapiens	sirtuin-3, mitochondrial isoform a	399	NP_036371
Homo sapiens	NAD-dependent protein lipoamidase sirtuin-4	314	NP_036372

Species	Description	Length (aa)	GenBank Accession no.
Homo sapiens	NAD-dependent protein deacylase sirtuin-5	310	NP_036373
Homo sapiens	NAD-dependent protein deacetylase sirtuin-6	355	NP_057623
Homo sapiens	NAD-dependent protein deacetylase sirtuin-7	400	NP_057622
Leishmania major	SIR2 homolog 1	373	Q25337
Leishmania major	SIR2 homolog 5	243	Q4Q2Y6
Leishmania major	SIR2 homolog 2	320	Q4QB33
Mycobacterium avium	SIR2 homolog 2	233	A0QC96
Mycobacterium avium	NAD-dependent deacetylase	282	ANR89928
Mycobacterium avium	SIR2 homolog	237	Q73WM7
Oryza sativa	SIR2-like protein	483	AAD42226
Pyrococcus abyssi	SIR2 homolog	250	Q9UZE7
Plasmodium falciparum	Sir2A	273	Q8IE47
Pyrococcus horikoshii	NAD-dependent deacylase	249	WP_010885040
Saccharomyces cerevisiae	Hst1p	503	AAA81033
Saccharomyces cerevisiae	Hst3p	447	AAA81034
Saccharomyces cerevisiae	Hst2p	357	AAA81035
Saccharomyces cerevisiae	Hst4	370	CAA88705
Saccharomyces cerevisiae	SIR2	562	CAA96447
Streptomyces coelicolor	SIR2 family transcriptional regulator	241	CAD55518
Streptomyces coelicolor	SIR2-like regulatory protein	299	NP_624772
Schizosaccharomyces pombe	sir2	475	O94640
Schizosaccharomyces pombe	SIR2 protein 4	415	Q9UR39
Schizosaccharomyces pombe	sir2 protein 2	332	Q9USN7
Salmonella typhimurium	SIR2 homolog	273	P0A2F2
Trypanosoma brucei	Sir2 homolog	351	AAC73004
Thermotoga maritima	regulatory protein, SIR2 family	246	AAD35575
Yersinia pseudotuberculosis	SIR2 homolog	278	Q8ZFR1

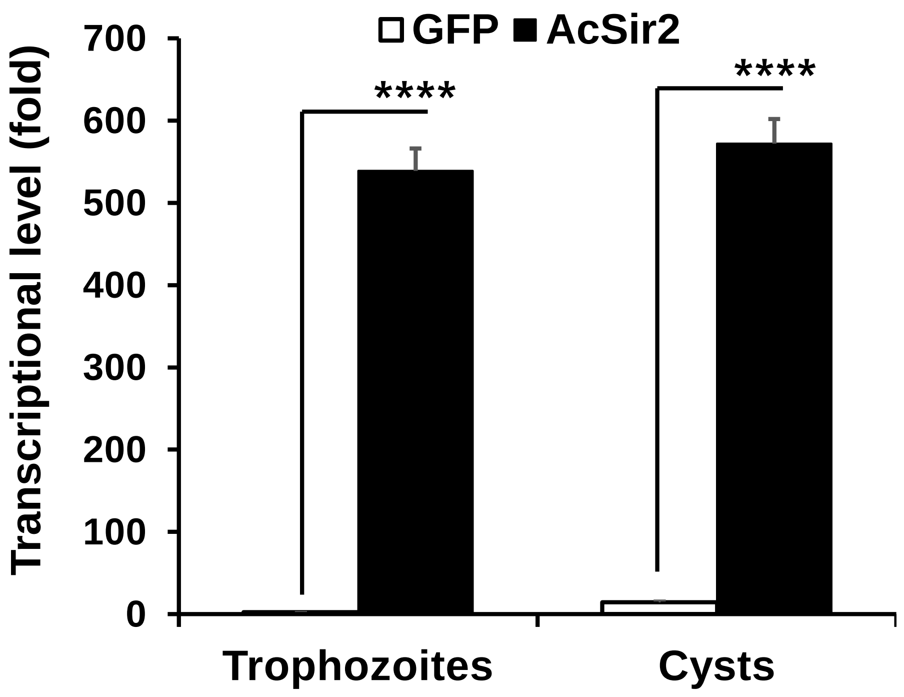

Additional file 1: Figure S3. Overexpression of AcSir2 in *A. castellanii* trophozoites and cysts. Trophozoites were transfected with pGAPDH-EGFP (light bars) or pGAPDH-AcSir2-EGFP plasmid (dark bars). A portion of the transfected trophozoites was transferred to encystation medium, incubated for 72 h, and examined for transcriptional changes in AcSir2 using qRT-PCR. The expression of AcSir2 was normalized to that of *Acanthamoeba* actin (**** $P < 0.0001$).

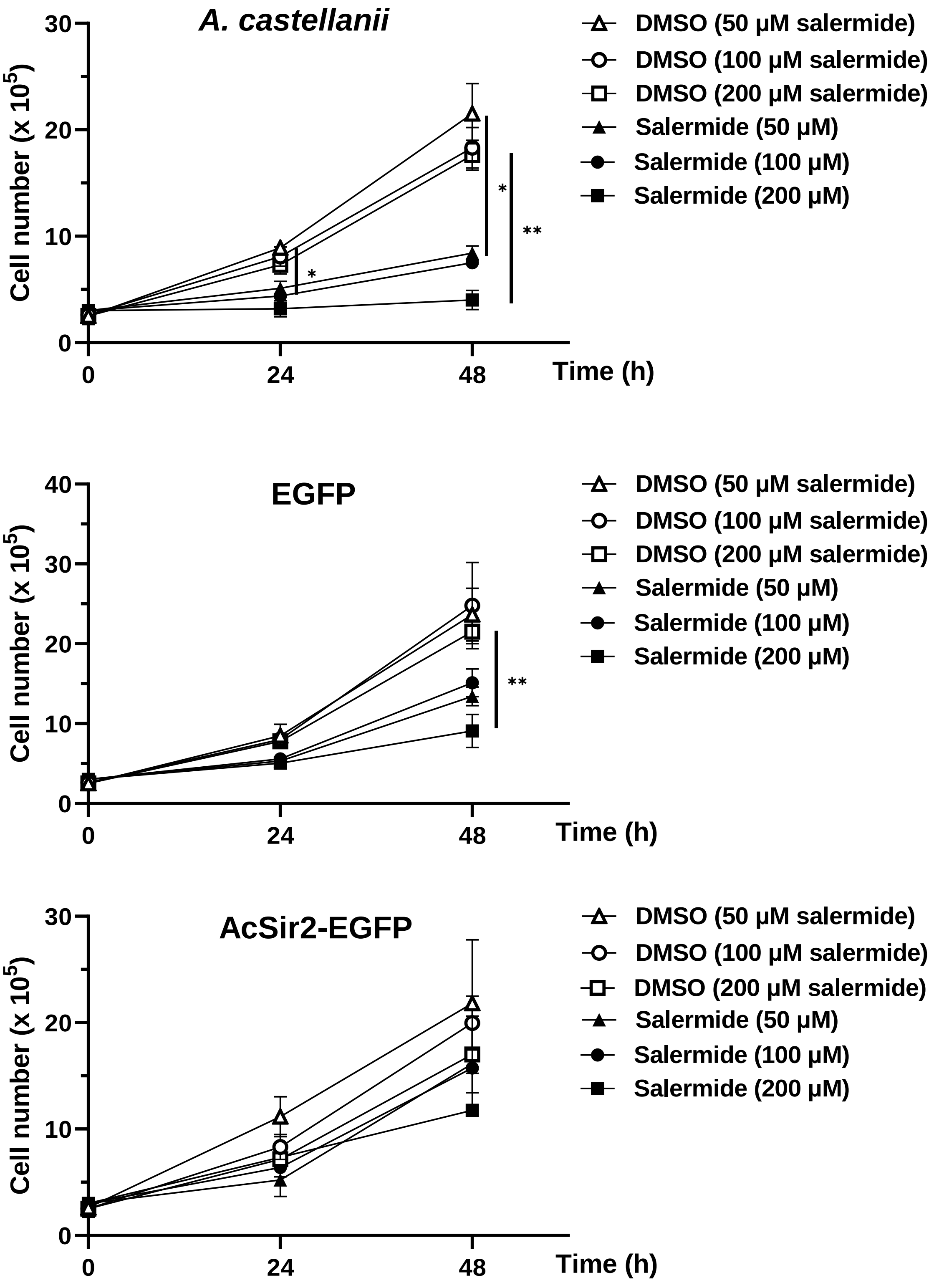

Additional file 1: Figure S4. Effects of salermide on proliferation of *A. castellanii* trophozoites (upper), and EGFP- (middle) and AcSir2-EGFP (lower) overexpressing trophozoites. Trophozoites were incubated with various concentrations of salermide or DMSO as a solvent control. Data represent mean cell numbers 0, 24, and 48 h after incubation for each salermide or DMSO concentration (*$P < 0.1$ and **$P < 0.01$).
